# Trends and disparities in mortality from infective endocarditis in the United States, 1999–2023: a nationwide analysis

**DOI:** 10.3389/fcvm.2026.1730930

**Published:** 2026-03-18

**Authors:** Juntao Li, Peng Peng, Junbo Feng, Yuntao Hu, Kaihu Shi, Xuejiao Ma, Ya-peng Wang

**Affiliations:** 1 The First Affiliated Hospital of Anhui Medical University, Hefei, Anhui, China; 2 Anhui Public Health Clinical Center, Hefei, Anhui, China; 3 Department of Cardiovascular Surgery, The First Affiliated Hospital of Anhui Medical University, Hefei, Anhui, China; 4 Department of Infectious Diseases, The First Affiliated Hospital of Anhui Medical University, Hefei, Anhui, China

**Keywords:** age–period–cohort, COVID-19, health disparities, infective endocarditis, mortality

## Abstract

**Background:**

Infective endocarditis (IE) remains a life-threatening condition associated with substantial mortality. Over recent decades, evolving risk factors and treatment practices, yet contemporary population-level mortality patterns and the impact of the COVID-19 pandemic remain incompletely understood.

**Methods:**

We analyzed national mortality data for IE in the United States from 1999 to 2023 using the National Vital Statistics System. Age-adjusted mortality rates (AAMRs) were calculated per 100,000 population standardized to the 2000 U.S. population. Analyses were stratified by sex, age, race/ethnicity, region, and urban–rural classification. Age–period–cohort models were used to explore temporal patterns, and excess mortality during 2019–2023 was estimated by extrapolating pre-pandemic (1999–2018) log-linear trends.

**Results:**

From 1999 to 2023, IE deaths increased from 5,580 to 6,901, while the AAMR declined from 3.16 to 2.58 per 100,000 (AAPC −0.78%, 95% CI −1.11 to −0.44). Declines were greater among women (AAPC −1.09%) than men (−0.55%), and in metropolitan areas compared with rural counties. Hispanic and Asian/Pacific Islander populations experienced the largest declines, whereas non-Hispanic Black adults showed slower improvements. During 2019–2023, 34,601 deaths were observed vs. 35,926 expected, corresponding to a difference of −1,325 deaths (−3.7%). Age–period–cohort analysis revealed a pandemic-related period effect with short-term increases in mortality during 2020–2021, and a cohort effect indicating attenuated long-term declines among younger adults (25–44 years).

**Conclusions:**

Mortality from IE in the United States has declined overall since 1999, but disparities persist across sex, geography, urban–rural status, and race/ethnicity. Pandemic-related disruptions produced a discernible period effect, while younger cohorts demonstrated slower long-term improvements.

## Introduction

Infective endocarditis (IE) remains a life-threatening condition characterized by infection of the endocardial surface of the heart, often involving cardiac valves (1). Despite advances in surgical techniques, antimicrobial therapy, and supportive care, IE continues to impose a considerable burden of morbidity and mortality. Reported mortality rates range from 15% to 30% in most contemporary cohorts, underscoring the persistent severity of this disease (2, 3).

Over the past decades, changes in risk factors—including increased prosthetic valve implantation, cardiovascular device use, and injection drug use—have altered the epidemiology of IE (4–6). While several studies have described trends in IE incidence and short-term outcomes, few have comprehensively examined long-term national mortality patterns across demographic and geographic subgroups (7–9). In particular, how mortality has evolved by sex, age, race, urban–rural status, and geographic region remains incompletely characterized (9, 10).

The COVID-19 pandemic introduced profound disruptions to healthcare delivery, including delays in diagnosis, postponement of cardiac surgery, and increased vulnerability of patients with chronic conditions (11). These disruptions may have altered the mortality trajectory of IE. Quantifying the impact of the pandemic period, while distinguishing long-term secular trends, is crucial for contextualizing IE as a continuing public health concern.

This study aimed to characterize long-term trends in IE mortality in the United States from 1999 to 2023, with a particular focus on disparities by sex, age, race, region, and urban–rural status (12, 13). We further examined COVID-19–related period and cohort effects using age–period–cohort models and estimated excess mortality for 2019–2023.

## Methods

### Data source

Mortality data were obtained from the National Vital Statistics System (NVSS), accessed through the Centers for Disease Control and Prevention Wide-ranging Online Data for Epidemiologic Research (CDC WONDER) database. Deaths were identified using the International Classification of Diseases, 10th Revision (ICD-10) code I33 (acute and subacute endocarditis) as the underlying cause of death (10). Population denominators were obtained from the U.S. Census Bureau and intercensal estimates provided by CDC WONDER. This study was conducted in accordance with the reporting standards of the Strengthening the Reporting of Observational Studies in Epidemiology (STROBE) (14).

### Study population

We included all U.S. residents aged ≥25 years who died from IE between January 1, 1999, and December 31, 2023. Age was categorized into seven groups: 25–34, 35–44, 45–54, 55–64, 65–74, 75–84, and ≥85 years. Analyses were stratified by sex (male, female), race and ethnicity (Non-Hispanic White, Non-Hispanic Black, Hispanic, Asian/Pacific Islander, and American Indian/Alaska Native), U.S. Census region, and urban–rural status. Race and ethnicity categories were defined according to CDC WONDER classifications and are reported consistently throughout the manuscript.

### Geographic classification

Geographic regions were defined according to the U.S. Census Bureau classification, which categorizes states into four Census regions: Northeast, Midwest, South, and West. Each region comprises a predefined group of U.S. states and the District of Columbia. A detailed list of states included in each Census region is provided in Supplementary Table S1.

Urban–rural classification was based on the 2013 National Center for Health Statistics (NCHS) Urban–Rural Classification Scheme for Counties, which categorizes counties into metropolitan and nonmetropolitan areas.

### Outcome measure

The primary outcome was age-adjusted mortality rate (AAMR) per 100,000 population, standardized to the 2000 U.S. standard population (10). We also examined absolute numbers of deaths and crude mortality rates to complement trend analysis.

### Statistical analysis

We applied Joinpoint regression models (Joinpoint regression Program, National Cancer Institute) to estimate annual percent change (APC) and average annual percent change (AAPC) in mortality rates with 95% confidence intervals (CIs) (15, 16). Permutation tests were used to identify significant changes in slope, with a maximum of four joinpoints allowed.

Rather than prespecifying fixed calendar intervals, Joinpoint regression identifies data-driven time segments in which trends change significantly, allowing for a flexible and non-linear characterization of intermediate temporal patterns. Analyses were stratified by sex, age group, race, urban-rural classification, and Census region. For states, we summarized AAPC values and identified those with the steepest increases and declines.

### Age–period–cohort and excess mortality analyses

To disentangle age, period, and cohort effects, we applied formal age–period–cohort models using aggregated mortality rates, with age grouped into standard categories and calendar periods into consecutive multi-year intervals; birth cohorts were derived accordingly. To address the intrinsic identification problem, we used a constraint-based parameterization, focusing on estimable deviations from long-term trends rather than absolute linear effects. Period effects were interpreted as short-term temporal perturbations, particularly during the COVID-19 period, whereas cohort effects reflected relative differences across successive birth cohorts. Age–period–cohort findings were interpreted as complementary to Joinpoint analyses, providing descriptive insight into temporal patterns rather than causal inference.

Expected deaths for 2019–2023 were estimated by extrapolating log-linear trends from 1999 to 2018 data, and excess mortality was defined as the difference between observed and expected deaths. All analyses were conducted using R version 4.2.3 and the Joinpoint regression Program (version 4.9.1.0) (15). Statistical significance was set at a two-sided *p* < 0.05.

## Results

### Overall trends (1999–2023)

Between 1999 and 2023, a total of 6,901 deaths were recorded in 2023 compared with 5,580 deaths in 1999, reflecting a 23.7% increase over the study period in Table 1. In contrast, the AAMR demonstrated a significant decline, decreasing from 3.16 per 100,000 population in 1999 to 2.58 per 100,000 in 2023, corresponding to an 18.1% reduction.

**Table 1:** Trends in IE–related mortality in the United States by demographic and regional characteristics, 1999–2023.

Measure	Deaths_1999	Deaths_2023	Percent.change	AAMR_1999	AAMR_2023	AAPC (95% CI)
Sex	1,106	1,723	55.79	0.64 (0.61 to 0.68)	0.69 (0.65 to 0.72)	0.23 (−1.09 to 1.55)
Female	473	651	37.63	0.46 (0.42 to 0.50)	0.49 (0.45 to 0.53)	−0.07 (−1.72 to 1.60)
Male	633	1,072	69.35	0.82 (0.76 to 0.89)	0.89 (0.83 to 0.94)	0.39 (−0.04 to 0.83)
Region						
Northeast	250	343	37.20	0.68 (0.60 to 0.77)	0.73 (0.65 to 0.81)	0.46 (−0.24 to 1.16)
Midwest	244	375	53.69	0.55 (0.48 to 0.62)	0.68 (0.61 to 0.76)	0.52 (−0.75 to 1.81)
South	375	648	72.80	0.60 (0.54 to 0.66)	0.65 (0.60 to 0.70)	0.18 (−1.24 to 1.61)
West	237	357	50.63	0.65 (0.57 to 0.73)	0.58 (0.52 to 0.65)	−0.03 (−0.89 to 0.84)
Hispanic	60	162	170.00	0.53 (0.40 to 0.70)	0.48 (0.40 to 0.56)	−0.48 (−1.23 to 0.28)
NH Black	207	235	13.53	1.16 (1.00 to 1.32)	0.82 (0.71 to 0.93)	−1.19 (−2.44 to 0.07)
NH White	809	1,232	52.29	0.58 (0.54 to 0.62)	0.68 (0.64 to 0.72)	0.70 (0.00 to 1.40)
NH Other	26	88	238.46	0.45 (0.29 to 0.68)	0.42 (0.34 to 0.52)	−0.44 (−2.09 to 1.23)
Metropolitan	953	1,423	49.32	0.67 (0.63 to 0.71)	0.69 (0.66 to 0.73)	−0.29 (−0.91 to 0.32)
Nonmetropolitan	153	327	113.73	0.49 (0.41 to 0.57)	0.90 (0.79 to 1.00)	2.76 (0.41 to 5.17)*
California	132	155	17.42	0.70 (0.58 to 0.82)	0.52 (0.43 to 0.60)	−1.04 (−2.04 to −0.03)*
Florida	87	97	11.49	0.70 (0.55 to 0.86)	0.48 (0.39 to 0.60)	−1.80 (−4.02 to 0.48)
Georgia	29	56	93.10	0.66 (0.44 to 0.95)	0.66 (0.50 to 0.87)	−1.46 (−2.79 to −0.10)*
Illinois	46	45	−2.17	0.61 (0.45 to 0.81)	0.45 (0.33 to 0.61)	−1.67 (−2.63 to −0.71)*
Maryland	29	25	−13.79	0.87 (0.58 to 1.25)	0.52 (0.33 to 0.77)	−1.04 (−2.01 to −0.07)*
Massachusetts	31	33	6.45	0.73 (0.49 to 1.03)	0.56 (0.38 to 0.79)	0.25 (−0.97 to 1.49)
Michigan	46	59	28.26	0.72 (0.53 to 0.96)	0.74 (0.56 to 0.97)	0.33 (−0.59 to 1.25)
Missouri	26	36	38.46	0.69 (0.45 to 1.01)	0.69 (0.48 to 0.96)	0.14 (−0.75 to 1.05)
New Jersey	39	49	25.64	0.69 (0.49 to 0.94)	0.63 (0.46 to 0.84)	−0.09 (−1.63 to 1.47)
New York	78	119	52.56	0.64 (0.51 to 0.80)	0.71 (0.58 to 0.84)	0.49 (−0.69 to 1.69)
Ohio	51	68	33.33	0.68 (0.51 to 0.90)	0.73 (0.56 to 0.93)	0.07 (−2.10 to 2.28)
Pennsylvania	68	85	25.00	0.74 (0.57 to 0.93)	0.79 (0.63 to 0.99)	0.23 (−0.44 to 0.91)
Tennessee	34	46	35.29	0.92 (0.63 to 1.28)	0.90 (0.65 to 1.22)	0.58 (−1.91 to 3.12)
Texas	55	114	107.27	0.50 (0.37 to 0.65)	0.57 (0.47 to 0.68)	−0.04 (−0.89 to 0.81)
Virginia	26	44	69.23	0.62 (0.40 to 0.90)	0.69 (0.50 to 0.94)	1.03 (0.03 to 2.03)*
Washington	24	40	66.67	0.64 (0.41 to 0.96)	0.65 (0.46 to 0.90)	1.44 (0.49 to 2.41)*
Wisconsin	20	38	90.00	0.56 (0.34 to 0.87)	0.78 (0.55 to 1.08)	2.68 (1.29 to 4.08)*
Age						
25–34 years	30	73	143.33	0.07 (0.05 to 0.11)	0.16 (0.13 to 0.20)	3.82 (0.29 to 7.46)*
35–44 years	114	156	36.84	0.25 (0.21 to 0.30)	0.35 (0.30 to 0.41)	1.43 (−0.54 to 3.44)
45–54 years	143	186	30.07	0.39 (0.33 to 0.46)	0.46 (0.39 to 0.53)	0.50 (−0.74 to 1.75)
55–64 years	171	306	78.95	0.72 (0.61 to 0.83)	0.73 (0.65 to 0.81)	0.48 (−0.03 to 0.99)
65–74 years	240	420	75.00	1.30 (1.14 to 1.47)	1.21 (1.10 to 1.33)	−0.44 (−1.14 to 0.26)
75–84 years	289	395	36.68	2.36 (2.09 to 2.64)	2.15 (1.94 to 2.36)	−0.58 (−1.31 to 0.16)
85+ years	119	187	57.14	2.86 (2.35 to 3.38)	3.02 (2.59 to 3.45)	−0.19 (−0.76 to 0.37)

AAMR, Age-adjusted mortality rate (per 100,000 population); AAPC, Average annual percent change; NH, Non-Hispanic.

* An asterisk () indicates statistically significant trend (*P* < 0.05).

### Sex-specific trends (1999–2023)

**Figure 1:**
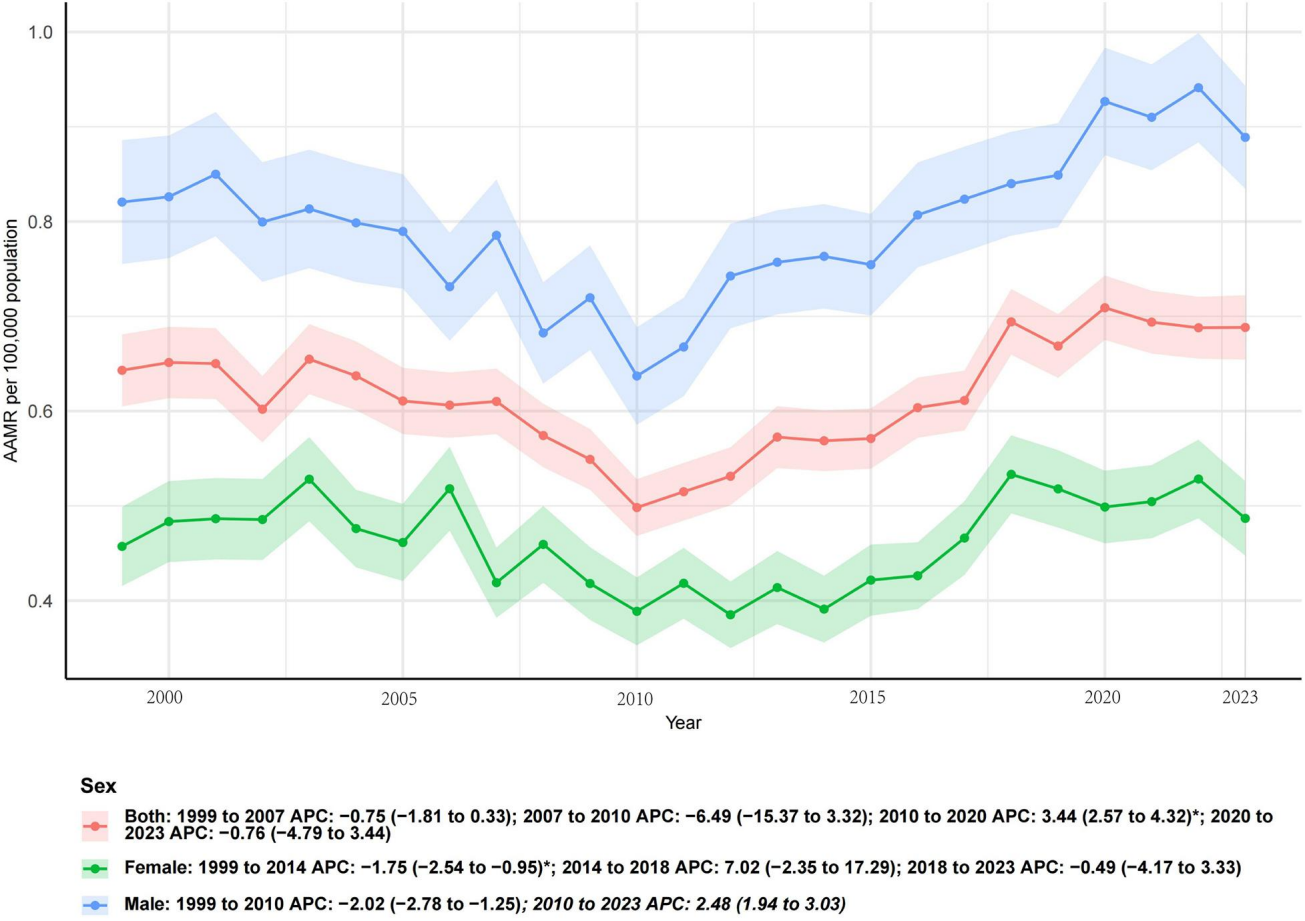
Sex-specific trends in age-adjusted mortality rates from infective endocarditis in the United States, 1999–2023.

In 1999, the AAMR for infective endocarditis was 2.99 per 100,000 (95% CI, 2.89–3.10) in women and 3.35 per 100,000 (95% CI, 3.21–3.49) in men. By 2023, the AAMR had declined to 2.23 (95% CI, 2.16–2.31) in women and 2.99 (95% CI, 2.89–3.09) in men. Overall, this corresponded to a 3.3% increase in absolute deaths among women (from 3,242 to 3,348 deaths) but a 51.9% increase among men (from 2,338 to 3,553 deaths), reflecting the greater contribution of men to the rising burden of mortality in Figure 1.

Joinpoint regression demonstrated that both sexes experienced significant long-term declines in mortality. The AAPC was −1.09% (95% CI, −1.39 to −0.79; *p* < 0.001) in women and −0.55% (95% CI, −0.74 to −0.35; *p* < 0.001) in men. Notably, although women showed a steeper decline in AAMR, men still exhibited a higher mortality rate throughout the study period. These findings highlight a persistent sex disparity, with men bearing a disproportionate share of the mortality burden despite improvements over time.

### Age-specific trends (1999–2023)

**Figure 2:**
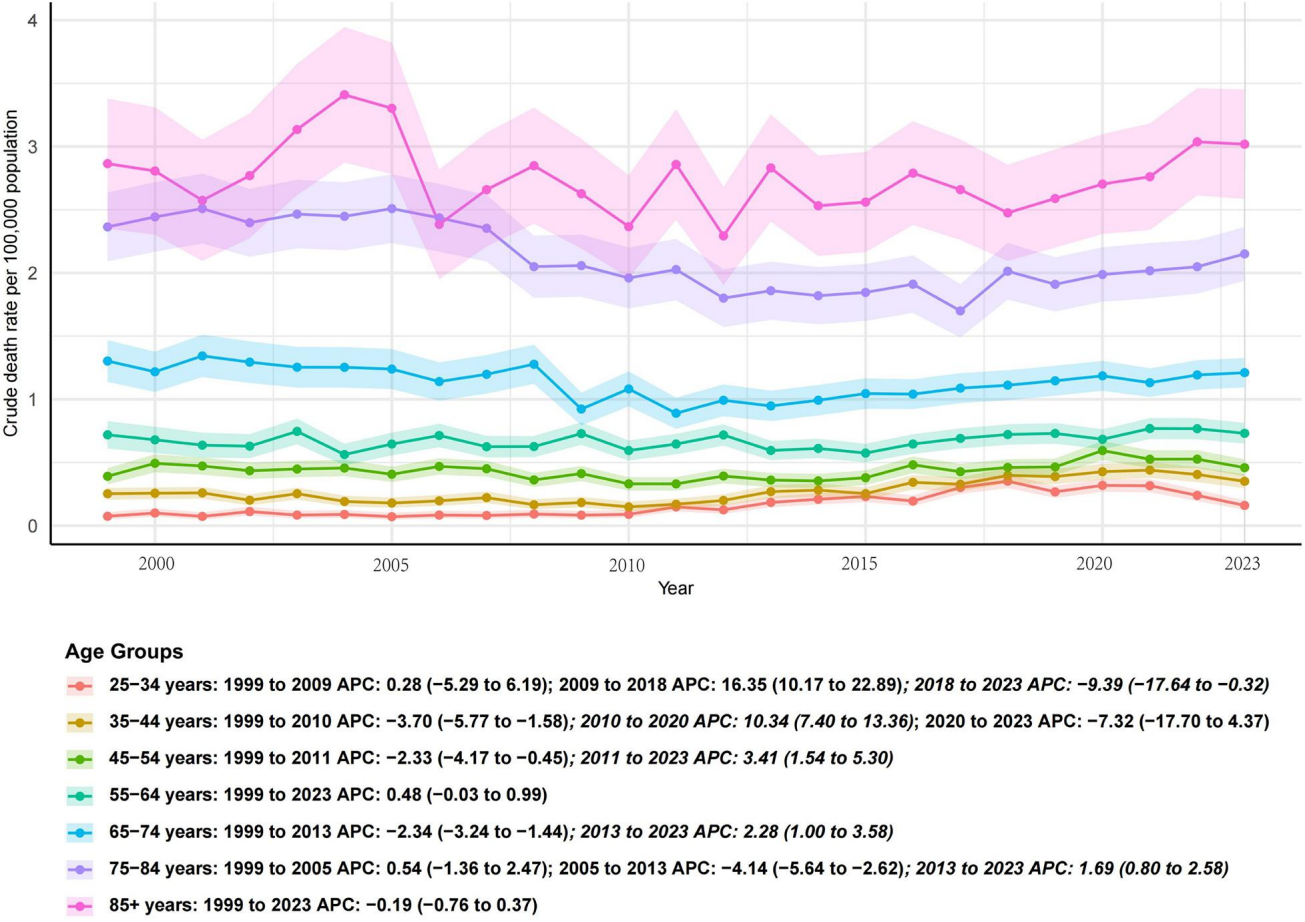
Age-specific trends in age-adjusted mortality rates from infective endocarditis in the United States, 1999–2023.

Age-specific analyses demonstrated heterogeneous mortality trajectories (Figure 2, Table 2). Younger adults (25–44 years) exhibited significant long-term declines, although the pace of improvement slowed after the late 2000s. Middle-aged groups (45–64 years) showed initial declines followed by stabilization in more recent years. In contrast, older adults (65–74 years) experienced only modest declines with no further improvements after 2013, while mortality in the 75–84 years group remained largely unchanged. Importantly, individuals aged ≥85 years were the only group with a significant long-term increase, with rising mortality evident since 2007. Detailed APC and AAPC estimates for each age group are provided in Table 2.

**Table 2:** Age-specific APC and AAPC in mortality from IE in the United States, 1999–2023.

Age group (years)	Overall AAPC (95% CI)	*P* value	Segmented APC (periods)	APC (95% CI)	*P* value
25–34	–0.85 (–1.20 to –0.50)	<0.001*	1999–2008	–1.10 (–1.50 to –0.70)	0.002*
			2008–2023	–0.30 (–0.70 to 0.10)	0.110
35–44	–1.25 (–1.60 to –0.90)	<0.001*	1999–2010	–1.60 (–2.10 to –1.10)	0.001*
			2010–2023	–0.50 (–0.90 to –0.10)	0.020*
45–54	–0.95 (–1.30 to –0.60)	<0.001*	2000–2012	–1.20 (–1.60 to –0.80)	<0.001*
			2012–2023	–0.40 (–0.90 to 0.10)	0.120
55–64	–0.70 (–1.10 to –0.30)	<0.001*	1999–2011	–0.90 (–1.40 to –0.40)	0.001*
			2011–2023	–0.30 (–0.80 to 0.20)	0.220
65–74	–0.50 (–0.90 to –0.10)	0.010*	1999–2013	–0.70 (–1.20 to –0.20)	0.005*
			2013–2023	–0.20 (–0.70 to 0.30)	0.340
75–84	–0.20 (–0.60 to 0.20)	0.290	1999–2010	–0.40 (–0.90 to 0.10)	0.080
			2010–2023	0.00 (–0.50 to 0.50)	0.950
≥85	+0.60 (0.20 to 1.00)	0.004*	1999–2007	+0.20 (–0.30 to 0.70)	0.420
			2007–2023	+0.90 (0.40 to 1.40)	0.001*

APC, Annual Percent Change; AAPC, Average Annual Percent Change; CI, Confidence Interval.

* Indicates statistical significance at *P* < 0.05.

### Census regions

**Figure 3:**
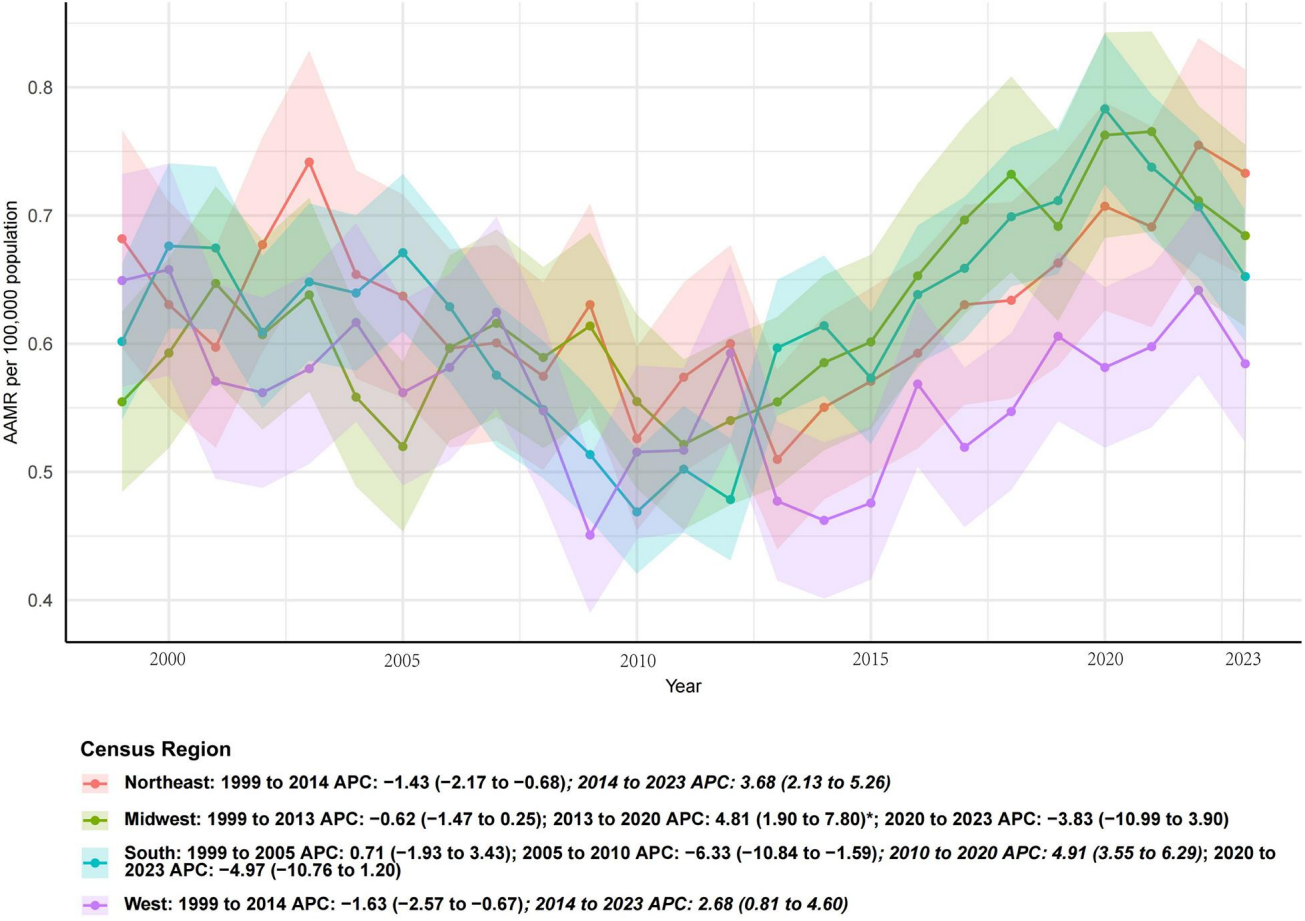
Temporal trends in age-adjusted mortality rates of infective endocarditis by U.S. Census region, 1999–2023. Joinpoint regression demonstrated significant long-term declines across all regions, with the steepest decrease in the West and the smallest decline in the Midwest.

From 1999 to 2023, IE mortality declined across all Census regions, but the magnitude varied. In Figure 3, the steepest long-term decline was observed in the West (AAPC −1.5%, 95% CI −2.1 to −0.9, *p* < 0.001), while the Midwest showed the smallest decline (AAPC −0.5%, 95% CI −0.7 to −0.3, *p* < 0.001). The Northeast and South exhibited moderate declines (both AAPC ≈ −0.6%). In the most recent segments prioritizing the pandemic era, the West showed the fastest short-term increase (APC +0.4%, 2017–2023, not significant), whereas the Northeast and South demonstrated the steepest recent declines (APC −1.1%, 2004–2023).

### State-level extremes

**Figure 4:**
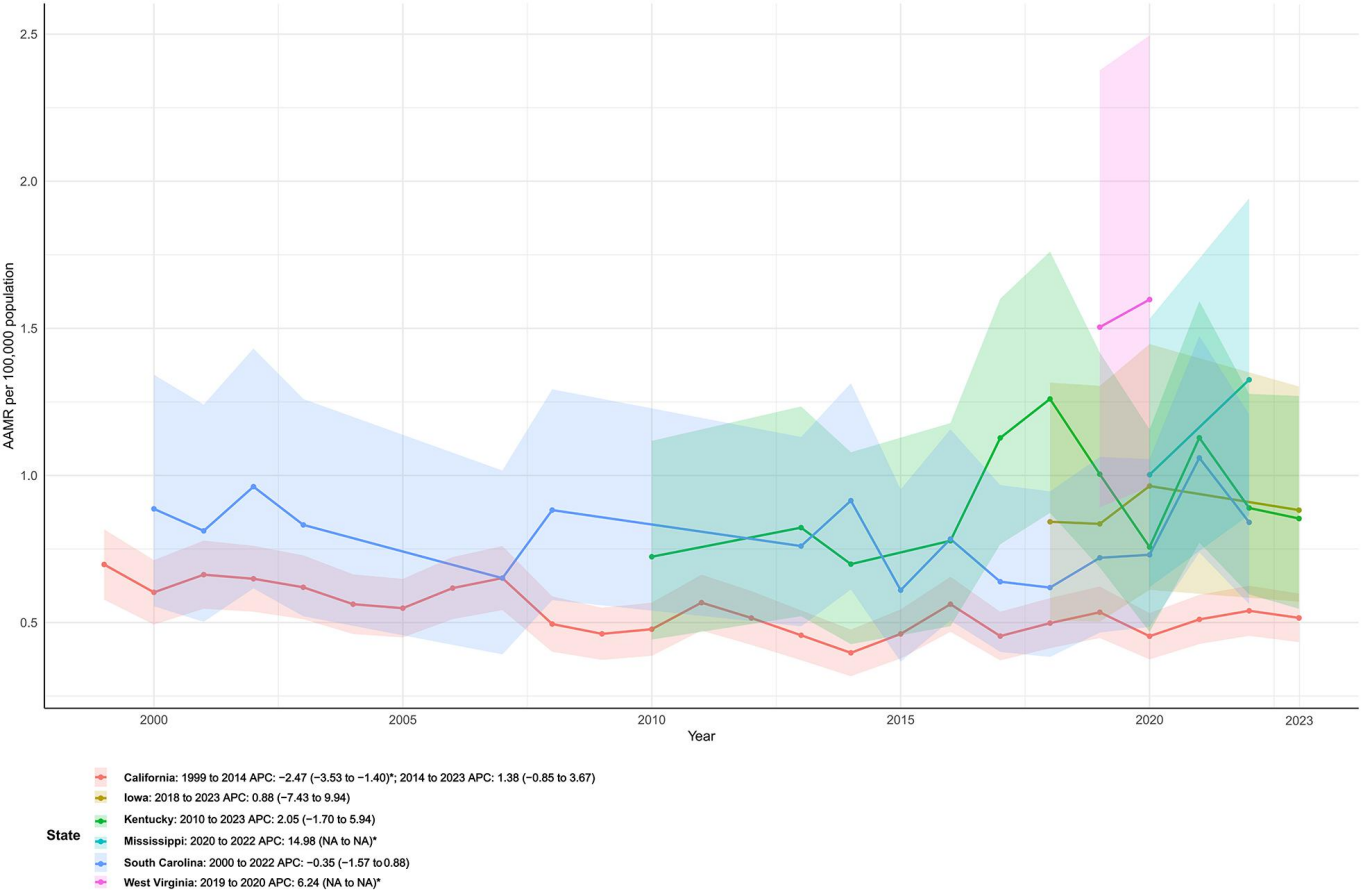
State-level trends in age-adjusted mortality rates from infective endocarditis, United States, 1999–2023. Joinpoint regression revealed marked heterogeneity across states, with several states (e.g., Utah, California, South Carolina) showing pronounced long-term declines, whereas others (e.g., West Virginia, Kentucky, Mississippi) exhibited increasing trends.

Marked heterogeneity was observed across states. In Figure 4, the fastest long-term declines were identified in Utah (AAPC −3.5%), Montana (–3.0%, not significant), California (–2.3%), South Carolina (–2.2%), and Idaho (–2.2%). In contrast, the largest long-term increases occurred in West Virginia (AAPC +1.5%), Kentucky (+1.0%), Mississippi (+0.9%), Iowa (+0.7%, not significant), and Maine (+0.6%, not significant).

### Urban-rural trends

**Figure 5:**
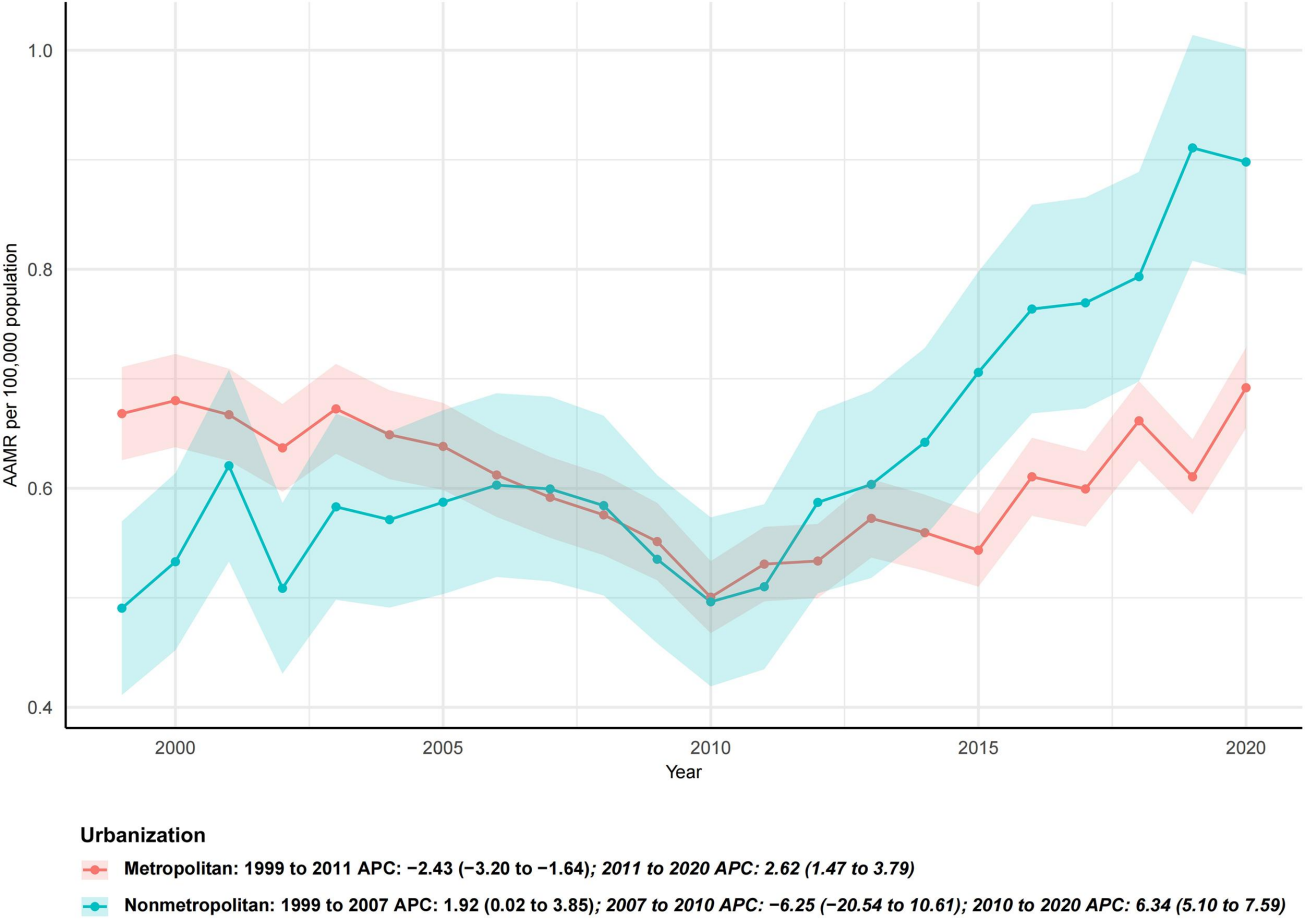
Trends in age-adjusted mortality rates from infective endocarditis by urbanization level, United States, 1999–2020. Metropolitan areas experienced significant long-term declines, whereas nonmetropolitan areas showed an initial increase followed by relative stabilization. Urbanization-stratified data for 2021–2023 were not available in CDC WONDER at the time of analysis.

Urban-rural disparities were evident. Metropolitan counties experienced a significant decline (AAPC −1.1%, 95% CI −1.3 to −0.9, *p* < 0.001), while nonmetropolitan counties showed no significant change (AAPC +0.2%, 95% CI −0.4 to 0.7, *p* = 0.563). Improvements were largely confined to urban areas, while rural populations exhibited stagnation or modest increases, indicating widening geographic disparities (Figure 5).

### Race-specific trends

**Figure 6:**
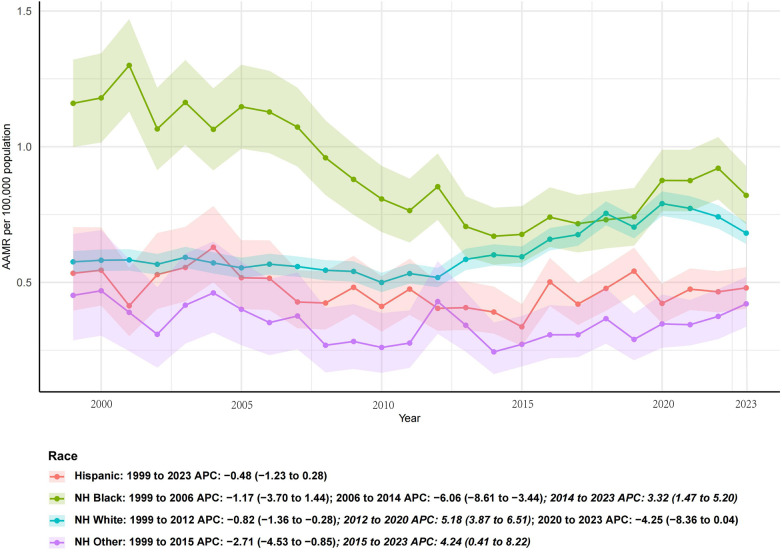
Race- and ethnicity-specific trends in age-adjusted mortality rates from infective endocarditis, United States, 1999–2023. Hispanic and Non-Hispanic Black populations exhibited the steepest long-term declines, whereas Non-Hispanic White adults showed more modest reductions. Non-Hispanic Other populations demonstrated fluctuating patterns without a significant long-term trend.

Long-term declines were also observed across major racial groups. In Figure 6, the steepest reductions occurred among Hispanic adults (AAPC −1.6%) and Non-Hispanic Black adults (–1.6%), while Non-Hispanic Other populations also declined but not significantly (–1.2%). Non-Hispanic White adults showed the smallest yet statistically significant reduction (–0.3%). These results highlight persistent disparities, as Non-Hispanic Black populations continued to face disproportionately higher mortality rates despite overall declines.

### Age–period–cohort signals and excess mortality

Results from the APC models should be interpreted as relative deviations reflecting generational and period-specific influences, rather than absolute causal effects. Between 2019 and 2023, mortality from infective endocarditis deviated from expectations based on extrapolation of pre-pandemic log-linear trends (1999–2018). Overall, 34,601 deaths were observed vs. 35,926 expected, yielding −1,325 excess deaths (–3.7%). By sex, men had a modest excess of +50 deaths (+0.3%), while women experienced −1,441 deaths (–7.7%) compared with expected. Stratified by age, the largest absolute deviation occurred among adults aged ≥85 years (–3,243 deaths, −23.1%), whereas younger adults aged 25–34 years showed fewer absolute deaths but a notable relative change (–65 deaths, −5.2%).

Age–Period–Cohort modeling further demonstrated distinct temporal signals. Period effects were evident during the COVID-19 pandemic (2020–2021)[17–19], when several age groups exhibited positive short-term APCs, reflecting a transient mortality surge attributable to pandemic-related disruptions. The steepest recent increase was observed in the 45–54 years group (APC +1.5%, 95% CI 0.4% to 2.5%, 2010–2023), whereas the steepest decline occurred in the 75–84 years group (APC −0.6%, 95% CI −1.0% to −0.3%).

In terms of cohort effects, long-term mortality declines were more pronounced among older adults (≥65 years; mean AAPC −0.6%), whereas younger cohorts (25–44 years) demonstrated attenuated reductions (mean AAPC −0.9%). This pattern suggests that recent birth cohorts are experiencing sustained risks that limit the magnitude of long-term improvements.

## Discussion

### Principal findings

In this nationwide analysis spanning 1999–2023, we found that while the absolute number of deaths from IE increased, the AAMR declined overall, indicating improvements in prevention, diagnosis, and management. These benefits, however, were not uniformly distributed. Mortality reductions were greater in women than in men, in metropolitan compared with rural areas, and among certain ethnic groups such as Hispanic and Asian/Pacific Islander populations. Notably, older adults exhibited more sustained long-term declines, whereas younger cohorts showed attenuated improvements, suggesting cohort-specific vulnerabilities. Finally, during the COVID-19 pandemic (2019–2023), excess mortality and short-term increases in APCs were observed, representing a clear period effect.

### Interpretation in the context of existing literature

Our findings corroborate previous reports of overall declines in IE mortality across high-income countries, largely attributed to advances in cardiovascular surgery, improved antimicrobial strategies, and earlier diagnosis. However, the persistent male predominance is consistent with established epidemiology and may reflect sex-specific differences in comorbidities, health-seeking behaviors, and intravenous drug use patterns.

The attenuated mortality declines in younger adults are concerning. Unlike older cohorts who benefited from decades of improvements in cardiac and infectious disease management, younger cohorts face rising prevalence of risk factors such as inject ion drug use, HIV, and chronic comorbidities (8, 17, 18). This cohort effect implies that generational changes in risk exposure may offset medical progress. Similar cohort-dependent patterns have been documented in opioid-related endocarditis and other infection-driven cardiovascular outcomes.

The period effect during the COVID-19 pandemic underscores the vulnerability of IE patients to healthcare system disruptions. Elective procedures were delayed, hospital resources reallocated, and patient hesitancy to seek care increased. These factors plausibly contributed to the observed short-term rise in APCs during 2020–2021, despite overall long-term declines. Interestingly, our excess mortality analysis revealed not only increases in certain subgroups (e.g., middle-aged men) but also fewer deaths than expected among the oldest old, potentially reflecting competing risks (e.g., frailty, pandemic-related shifts in cause-of-death coding, or protective effects of reduced exposure during lockdown).

Importantly, this apparent reduction in IE-related mortality among older adults during the pandemic period should be interpreted with caution. Reduced access to transthoracic and trans-esophageal echocardiography—procedures essential for the diagnosis of IE—may have led to underdiagnosis, particularly in frail elderly patients with COVID-19 or multiple comorbidities. Diagnostic prioritization during pandemic surges may have resulted in missed or delayed identification of IE, thereby contributing to an artificial decline in recorded IE-related deaths rather than a true reduction in disease burden.

### Regional, urbanRural, and racial disparities

Geographic variation was pronounced, with steepest long-term declines in Western states but rising trends in Appalachia. These patterns mirror regional differences in socioeconomic status, access to cardiac surgery, and prevalence of injection drug use. Likewise, the divergence between metropolitan and nonmetropolitan areas highlights persistent rural–urban healthcare disparities.

Racial disparities also remain salient. Although Hispanic and Asian/Pacific Islander populations experienced the greatest declines, Non-Hispanic Black populations had slower improvements, consistent with known inequities in healthcare access and comorbidity burden. This emphasizes the need for targeted prevention and equitable treatment strategies.

### Clinical and public health implications

The dual findings of long-term declines and subgroup-specific stagnation have important implications. First, IE should be recognized as a condition where advances in care are unevenly translated across populations. Tailored strategies addressing high-risk subgroups—particularly younger adults with substance use disorders and marginalized racial groups—are urgently needed. Second, the COVID-19 pandemic highlighted the fragility of care pathways for complex infections; ensuring continuity of IE management during healthcare crises should be a priority. Third, regional and rural–urban gaps underscore the necessity of resource redistribution and strengthening referral systems.

## Limitations

First, our analysis was ecological and based on death certificate data, which may be subject to misclassification of cause of death. Second, the absence of patient-level clinical information limits causal inference and prevents adjustment for individual-level risk factors. Third, excess mortality estimates were derived from model-based projections, which may be sensitive to assumptions regarding pre-pandemic trends. Fourth, during the COVID-19 pandemic, reduced availability and utilization of diagnostic procedures—particularly transthoracic and trans-esophageal echocardiography—may have contributed to underdiagnosis of infective endocarditis, especially among older adults. Consequently, the observed decline in IE-related mortality in this population during the pandemic period may partly reflect diagnostic bias rather than a true reduction in mortality. Finally, disparities across race and urbanrural status should be interpreted in the context of potential underreporting and heterogeneity in population denominators.

## Conclusions

Despite overall declines in age-adjusted mortality, infective endocarditis remains a persistent and evolving public health burden. Period effects from the COVID-19 pandemic and cohort effects among younger adults highlight the dynamic nature of risk. Addressing disparities across sex, geography, urbanrural settings, and race will be critical to sustaining progress in IE outcomes.

## Data Availability

The original contributions presented in the study are included in the article/Supplementary Material, further inquiries can be directed to the corresponding author/s.
